# DNA identification by pedigree likelihood ratio accommodating population substructure and mutations

**DOI:** 10.1186/2041-2223-1-8

**Published:** 2010-10-04

**Authors:** Jianye Ge, Bruce Budowle, Ranajit Chakraborty

**Affiliations:** 1 Department of Forensic and Investigative Genetics, University of North Texas Health Science Center, Ft Worth, Texas 76107, USA; 2 Institute of Investigative Genetics, University of North Texas Health Science Center, Ft Worth, Texas 76107, USA

## Abstract

DNA typing is an important tool in missing-person identification, especially in mass-fatality disasters. Identification methods comparing a DNA profile from unidentified human remains with that of a direct (from the person) or indirect (for example, from a biological relative) reference sample and ranking the pairwise likelihood ratios (LR) is straightforward and well defined. However, for indirect comparison cases in which several members from a family can serve as reference samples, the full power of kinship analysis is not entirely exploited. Because biologically related family members are not genetically independent, more information and thus greater power can be attained by simultaneous use of all pedigree members in most cases, although distant relationships may reduce the power. In this study, an improvement was made on the method for missing-person identification for autosomal and lineage-based markers, by considering jointly the DNA profile data of all available family reference samples. The missing person is evaluated by a pedigree LR of the probability of DNA evidence under alternative hypotheses (for example, the missing person is unrelated or if they belong to this pedigree with a specified biological relationship) and can be ranked for all pedigrees within a database. Pedigree LRs are adjusted for population substructure according to the recommendations of the second National Research Council (NRCII) Report. A realistic mutation model was also incorporated to accommodate the possibility of false exclusion. The results show that the effect of mutation on the pedigree LR is moderate, but LRs can be significantly decreased by the effect of population substructure. Finally, Y chromosome and mitochondrial DNA were integrated into the analysis to increase the power of identification. A program titled MPKin was developed, combining the aforementioned features to facilitate genetic analysis for identifying missing persons. The computational complexity of the algorithms is explained, and several ways to reduce the complexity are introduced.

## Background

Over the past two decades, forensic DNA typing has become widely accepted as a powerful tool in criminal and civil investigations. This technology has become invaluable in many missing-person identifications. There are a number of scenarios in which person identification is required: these include cases of war victims found in mass graves, missing soldiers or military personnel from past wars, people missing due to dynamic social reasons (for example, murder), remains from mass disasters due to natural catastrophes or terrorism attacks (for example, airplane crashes, the World Trade Center tragedy and the southeast Asia tsunami) and basic paternity testing. In attempts to identify these individuals, DNA profiles from unidentified people may be compared with direct reference samples of the missing person (antemortem samples), such as buccal swabs collected before their disappearance, or items they have used, such as toothbrushes, hairbrushes or preserved dental casts. In some cases, direct comparisons are not possible because an antemortem sample is not available, or the chain of custody may not be established reliably, reducing the confidence in an association. Alternatively, a missing person may be identified by kinship analysis using family reference samples (biological relatives such as parents, offspring, siblings or cousins) of the person to be identified.

Traditionally, statistical inference was based on pairwise comparison of the DNA profiles of the unknown sample and a single family reference sample, and then ranking the likelihood ratios (LRs) for specified biological relationships. Numerous statistical methods are available for evaluation of kinship between individuals. Li and Sacks [1] first provided a general method to obtain the conditional probability for any pair of relatives. Jacquard [2] described the most general method for a pairwise relationship using nine condensed identity states. Thompson [3] pioneered the maximum likelihood method by summarizing the k coefficients for major pairwise relationships, which were the probabilities that two individuals might have 0, 1 or 2 genes identical by descent. However, a pairwise comparison does not exploit the potential full power for identification, because it does not take into account all genetic information jointly when multiple family reference samples are available. Substantial progress in the past few years has been made in the determination of missing-person identity by pedigree kinship analysis [4-8]. Lau *et al. *[8] used standard parentage analysis, which includes both parents of a missing person, for the identification of victims of the Indian Ocean tsunami disaster of 2004. Buckleton *et al. *[9] discussed pedigree LR calculations with adjustments for population substructure effects with a few simple examples; however, no detailed algorithm for pedigree LR was given. Drabek [10] reviewed current software used for kinship analysis and reported that a number of software programs can provide the function to calculate pedigree likelihoods but they do not all offer comprehensive approaches. Dawid *et al. *[11] used a Bayesian network for identification using pedigree information, which incorporated the possibility of mutation, but with no adjustment for population substructure. DNAView [12] can calculate pedigree LRs without population substructure correction. For simple paternity cases, a short tandem repeat (STR) mutation model was implemented, which requires users to specify how rare it is for a mutational event of ≥ 2 steps to occur. For complex pedigrees (kinship), DNAView implemented an 'AABB' model, which simply assigns 'PI = μ', where μ is the locus-specific mutation rate. As stated in the DNAView manual [13], this model is 'a very crude way' (page 92) and could lead to 'a gross underestimate' (page 110). Hepler *et al. *[14] did incorporate population substructure into HUGIN (Handling Uncertainty In General Inference) but did not address mutation. Familias [15] does address both population substructure and mutation, but the mutation models are not appropriate for human STR loci. The 'equal probability model' and 'proportional model' used in Familias are not necessarily the best for STR loci [16,17], and the 'decreasing model' includes a parameter (number of 'possible' alleles) that cannot be determined, because mutation probability is not related to allele frequency and the number of possible alleles [16,17].

In all the above approaches, the details of genotype inference for the untyped family members in the reference pedigree were not disclosed, especially when both population substructure and mutation were incorporated. The computational complexity of the pedigree LR was not presented, and only autosomal loci were considered in the identification calculation. In addition, it has not been recognized in these studies that the mutation rates for generating integer and fractional STR alleles are different.

In this study, we combined pedigree analysis, population substructure and mutation analysis, and developed a method to calculate pedigree LR based on the classic Elston-Stewart (ES) algorithm [18]. To facilitate the use of the described pedigree analysis, a software program (MPkin) was developed. Population substructure was incorporated to comply with recommendation 4.1 in the NRCII Report [19]. A realistic mutation model is also embedded to address potential mismatches between true biological relatives, so that the method will yield a LR for any pedigree, although the number could be very small for pedigrees with multiple large-step mutations. Ge *et al. *[20] previously described the basic idea of the method to calculate pedigree LR with examples in absence of population substructure and mutation. Because reference family member(s) may not be available to type, the details of the methods used to infer the genotypes of untyped references were discussed. The theoretical computation complexity for pedigrees with inferred genotypes for untyped family members was analyzed. Several approaches were introduced to reduce the exponential computation complexity caused by untyped individuals in a family pedigree. The computational complexity of pedigree likelihood ratio (PLR) calculations with population substructure and/or mutations was compared. In addition, calculation of LRs for Y chromosome haplotypes and mitochondrial (mt)DNA haplotypes was performed, which can be directly combined with LR of autosomal STRs under the assumption of independence.

## Method

### General principle

To evaluate whether a missing person (*MP) *belongs to a family pedigree (*P*), one or more reference family members from the putative pedigree are typed. Identification is assessed by comparing two alternative hypotheses: (i) H_p_: *MP *is the specific member of the putative pedigree and (ii) H_d_: *MP *is unrelated to the known reference members of the putative pedigree.

The LR is calculated based on probability of the DNA evidence under each hypothesis, represented by the general expression:

(1) $$LR=\frac{\mathrm{Pr}(G_{MP},G_{P}|H_{p})}{\mathrm{Pr}(G_{MP},G_{P}|H_{d})}$$

where *G_MP _*refers to the DNA profile of the missing-person (from remains) and *G_P _*is the joint DNA profile of all typed family members in the pedigree, computed conditions imposed by the hypotheses H_p _and H_d_, respectively. H_p _is favored if the LR is > 1; when the LR is < 1, H_d _is better supported. For H_p_, the position of *MP *in *P *is usually fixed. However, several scenarios could apply to H_d_; for example, the biological mother but not biological father of *MP *is already in *P*, *MP *is a half sibling but not a full sibling of someone in *P*, or *MP *is not related to anyone in *P*. Multiple LRs can be compared in terms of different H_d_. If no prior information of *MP *is provided to specify H_d_, *MP *may be regarded as not related to anyone in *P*.

### Pedigree likelihood algorithm

The ES [algorithm 18] calculates the probability by 'peeling' the pedigree into multiple nested nuclear families. In brief, the ES algorithm can be adapted to the likelihood of a pedigree as:

(2) $$L=\sum_{G_{1}}...\sum_{G_{n}}\mathrm{Pr}(G_{founder})\prod_{founder}\prod_{\left\{o,f,m\right\}}\mathrm{Pr}(G_{o}|G_{f},G_{m}),$$

in which *G_i _*represents the genotype (at a specific locus) of the *i*-th person of a pedigree, and each member is classified as either a founder (that is, a person without antecedent relatives in the pedigree, with their genotype represented as *G_founder_*), or an offspring (*G_o_*) from a given mother (*G_m_*) and father (*G_f_*). The locus-specific likelihood (*L*) of a pedigree is the summation over all possible genotype combinations, *G_i_*, for each member (of course, for the typed members in the pedigree, the observed genotypes are considered as the only possibility). Within the summation, the probability of each possible genotype combination of a pedigree is computed as the product of two factors: (i) joint probability of all founder genotypes, *Pr(∏G_founder_) *and the product of each of the probabilities of offspring genotypes conditional on parental genotypes for trios, Pr(*G_o_*|*G_f_*, *G_m_*) or (ii) the probability of allele transmission in the pedigree. Computed in this fashion, values for *L *across all loci are multiplied to get a combined *L *value, denoted by *Pr(G_MP_, G_P_)*, which is in turn used in the final LR calculation (see equation 1). The computational complexity of the ES algorithm increases linearly with the number of trios in a complete pedigree (that is, a pedigree with all family members typed).

Using this algorithm as the general rule of pedigree likelihood evaluation, under the hypothesis *H_p_*, evaluation of *Pr(G_MP_, G_P _*|*H_p_) *in equation (1) is performed with the genotype, *G_MP_*, of the missing person (from remains) as the genotype of their presumed position in the pedigree. By contrast, under the hypothesis *H_d_*, *MP *is simply an unrelated individual to any other reference family members.

### Genotype inference of untyped persons

In some situations, not all family members of a reference pedigree may be typed. Genotypes of these untyped individuals can only be inferred from those of the typed relatives, such as parents (one or both of them typed), offspring and spouse, under the assumption that the untyped family members are truly the designated biological relative specified. For example, for two parents with the genotypes {11, 12} and {13, 14}, the possible genotypes of their offspring, barring mutations, are {11, 13}, {11, 14}, {12, 13} and {12, 14}. Likewise, without any mutation, given a mother that has genotype {11, 12} with two of her offspring being {11,13} and {11,14}, the biological father of the children is inferred as {13,14}.

If a mutation is a possible consideration even for individuals with typed parents or offspring, the genotype of untyped individuals theoretically can be all possible genotypes at that locus. However, not all genotypes need to be inferred for each untyped individual in the pedigree. The computational complexity can be reduced by reducing the number of individuals with inferred genotypes. For nuclear families with a single offspring and a single typed parent, the genotypes of the untyped parent are not needed. For nuclear families with several offspring, the genotypes of both parents should be inferred, because the probabilities of allele transmissions from the untyped parent to multiple offspring are not independent. For example, for a family with a single typed parent {10, 11} and two offspring {10, 12} and {10, 13}, the transmission probability is not equal to 1/2*Pr(12)*1/2*Pr(13). The preferred approach is to infer the genotype of the untyped parent, {12, 13} and then calculate the transmission probability (1/16) based on the genotypes of both parents.

To reduce computational complexity, an untyped individual, defined as one whose genotypes has to be inferred, is termed an untyped connector (UC), which includes (1) untyped founders with > 1 offspring, (ii) untyped founders with a single offspring but an untyped spouse, (iii) untyped non-founders who are not leaf or bottom nodes in the pedigree tree.

**Figure 1:**
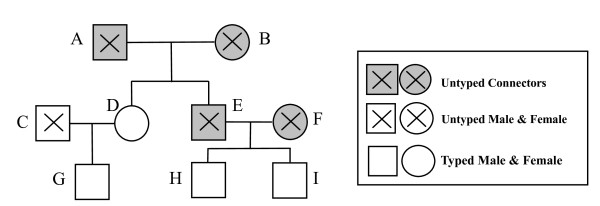
**Family pedigree**. A, B, C, E and F are untyped family members; D, G, H and I are typed members. *Untyped Connectors (UC) *include A, B, E and F. G is an *Offspring with Single Typed Parent *(*OSTP*).

For nuclear families with single offspring, one untyped parent, and one typed or UC parent, the single offspring is defined as an 'offspring with single typed parent' (OSTP). OSTPs are important in population substructure adjustment because the transmitted allele from the typed or UC parent is undecided. Figure 1 gives an example to illustrate both definitions. A, B, E and F in this example are UCs, and G is an OSTP. The genotype of C does not need to be inferred because there is only one offspring G in the nuclear family {C, D, G} (Figure 1).

### Population substructure correction

Population substructure induces a degree of correlation of uniting gametes in randomly chosen individuals from the population. Hence, population substructure corrections for the probability calculations were recommended by previous publications [19,21]. This correlation is measured by the co-ancestry coefficient (*θ*), that is, the probability that random sampled alleles from two individuals are identical by descent. The probability that an allele *A *will be observed, given that *x *alleles of type *A *have been observed in all observed *n *alleles,

(3) $$\text{is}\mathrm{Pr}(A|\text{Observed Alleles})=\frac{x\theta +(1-\theta )p(A)}{1+(n-1)\theta }$$

where *p(A) *is the allele frequency of allele *A *[21,22]. According to the NRCII recommendation, *θ *is set at 0.01 for large populations and 0.03 for small, isolated populations, but can be set to population- and even locus-specific *θ *values.

The likelihood of founder alleles can be calculated by selecting all founder alleles one by one based on formula (3). For example, the likelihood of two typed founders, {A, B} and {C, D}, is

(4) L=Pr(A)Pr(B|A)Pr(C|AB)Pr(D|ABC)

The probability of transmission from parents to offspring {E, F} is calculated as shown in equation 5, if both parents are typed [so *Pr(X > Y) *= 1 if allele *X *and allele *Y *are identical by descent, otherwise in the absence of a mutation it is 0].

(5) $$\begin{array}{r} P(EF|AB,CD)=1/2*[\mathrm{Pr}(A\to E)+\mathrm{Pr}(B\to E)]\\ {}*1/2*[\mathrm{Pr}(C\to F)+\mathrm{Pr}(D\to F)]\\ +1/2*[\mathrm{Pr}(A\to F)+\mathrm{Pr}(B\to F)]\\ {}*1/2*[\mathrm{Pr}(C\to E)+\mathrm{Pr}(D\to E)] \end{array}$$

For cases with a single typed parent {A, B} and a typed offspring {E, F}, transmission likelihoods need to be calculated with caution, because the allele transmitted from the parent is undetermined, that is, either E or F could be the transmitted allele or founder allele. If there is only one OSTP in the pedigree, two possible scenarios are considered: E is transmitted from the typed parent and F is the founder allele, and *vice versa*. The transmission probability within the trio is based on the summation of transmission probabilities for both scenarios.

(6) $$\begin{array}{r} L=1/2*[\mathrm{Pr}(A\to E)+\mathrm{Pr}(B\to E)]\mathrm{Pr}(F|AB)\\ +1/2*[\mathrm{Pr}(A\to F)+\mathrm{Pr}(B\to F)]\mathrm{Pr}(E|AB) \end{array}$$

If there is > 1 OSTP in the pedigree, all possible transmission patterns are considered, and the transmission likelihood of the pedigree is calculated by summarizing likelihoods of all transmission patterns. For a pedigree with all genotypes of UCs assigned, *n *(number of OSTPs in the pedigree) generates *2^n ^*possible patterns, and pedigree likelihoods can be calculated by

(7) $$L=\sum_{O_{1}}...\sum_{O_{n}}\mathrm{Pr}(Pedigree|O_{1},...O_{n})$$

where *O_i _*is the *i-*th OSTP. Each *O_i _*has two possibilities: the first or the second allele is a founder allele. In this situation, the likelihood of founders and the likelihood of transmission cannot be clearly separated, because they are not independent.

### Mutation correction

Mutations are genetic alterations that may occur during transmission of alleles from parent to offspring. If not considered, a mutation can lead to false exclusion because of a difference at the obligate allele between two related individuals. There are several theoretical mutation models for different types of markers and genetic assumptions, such as the Two Phase Model [23-25], the Infinite Allele Model [26], the Stepwise Mutation Model [27] and the K-Allele Model [28]. The most applicable one for most human STR or microsatellite markers is the Two Phase Model [25], which is a symmetrical mutation model allowing alleles to change by adding or subtracting an absolute number of *x *repeat units. The transmission probability of two identical allele is 1- μ. The probability of a mutation event with *x *step (*x *> 0) is

(8) $$\mathrm{Pr}(X=x)=\mu \alpha {\left(1-\alpha \right)}^{x-1}$$

where *α *is the probability of being a one step mutation and *μ *is the mutation rate of the locus. Equal probabilities for gaining or losing repeats are assumed.

According to the AABB annual report [29], > 95% of mutations result in one-step differences, hence *α *was set at 0.95; mutations of > 2 steps are unlikely, but several mutation steps are allowed in this model. The mutation rates of the forensically used STR loci are on the order of 10^-3 ^to 10^-4 ^per locus per generation [29,30]. Moreover, as the number of members in a pedigree and the number of STR loci used for analysis increase, the chance of detecting a mutation increases. Hence, the potential for mutation must be accommodated. Moreover, because males have higher mutation rates than females [29], different locus-specific mutation rates must be used for the father and mother within a pedigree.

The mechanism of mutations between integer (for example, 9) and fractional (for example, 10.2) STR alleles is different from slippage-based mutation. The probability of a partial repeat mutation should be lower than the average STR mutation rates and higher than the SNP mutation rates (for example, 10^-8^). Because there are no data on partial repeat mutations, we arbitrarily set the probability at 10^-5^, but further investigations are needed to establish a more meaningful probability.

### Y chromosome and mtDNA

Autosomal STRs, Y chromosome haplotypes and mtDNA haplotypes do not display departures from expectations of independence, except when notable levels of substructure were detected [31,32]. Hence, LRs of Y and mtDNA haplotypes can be directly combined together and with LRs for autosomal STRs. The general principle of calculating LRs of Y and mtDNA haplotypes is the same as that for autosomal markers, which compares the likelihoods of missing-person and putative pedigree haplotypes given H_p _(that is, the product of multiple haplotype transmission probabilities) or H_d _(that is, haplotype frequency in a population).

There may be multiple Y or mtDNA references in a putative pedigree. Only the closest available relatives are considered, because minimum transmission reduces the probability of mutations. The number of haplotype transmissions is used to determine the closest relatives. For example, if Y haplotypes are available for the father and uncles of a putative missing person, only the haplotype of the father is considered. Father-offspring is the closest relationship for Y chromosome markers with only one haplotype transmission; followed by full siblings, grandchildren or grandparents. For mtDNA haplotypes, the same logic applies to the maternal lineage. The transmission probability between Y haplotypes is the product of the transmission probability of alleles at each locus under the Two Phase Mutation Model. For mtDNA haplotypes, the transmission probability of two mtDNA haplotypes with > 2 nucleotide differences is 0; otherwise, it is 1 [33].

## Discussion

### Computational complexity analysis

The computational complexity of a pedigree LR calculation generally depends on the number of markers (*NM*), the number of UCs (*NUC*), the number of possible genotypes of each UC (*NGUC*) and the number of OSTPs (*NO*). The complexity or the number of pedigrees with genotypes of UCs assigned, can be presented as

(9) $$NM*\prod_{i=1...NUC}NGUC_{i}*2^{NO}$$

The genotypes are not inferred for all untyped individuals but OSTP is defined because NGUC is always > 2 with the possibility of mutation. By OSTP, the computation could be several orders of magnitudes faster for large pedigrees with several untyped individuals. Thus, *NUC *and *NGUC *make up the major contribution for complexity. Without mutation, *NGUC *is small compared with the number of all possible genotypes. However, with the presence of mutation, *NGUC *is close to its maximum possible number. One approach to reduce *NGUC *is to summarize all alleles that were not observed in the pedigree as a new allele '*X*'. The frequency of '*X*' is the complement of the sum of frequencies of all possible present alleles, including possible mutated alleles.

(10) $$p(X)=1-\sum_{\text{Possible present alleles}}p(allele)$$

If a pedigree can be separated into several independent subpedigrees, the complexity will be further reduced by using one multiplication between likelihoods of two subpedigrees instead of several multiplications for several *NGUC *values. For many cases of likelihood calculations for a given H_d_, this partition could significantly reduce the complexity.

**Figure 2:**
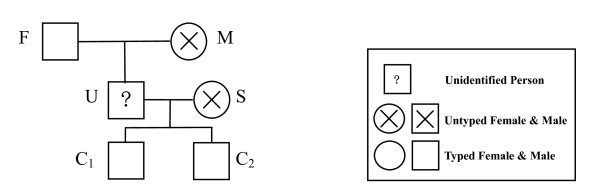
**Pedigree to identify the missing person U**. In this, C1 and C2 are the alleged children of U, F is alleged father, M is the alleged mother and S is the alleged spouse. M and S are not typed. S is an UC, whose genotype has to be inferred.

If all loci of all references are typed, the complexity reduces to *NM*, and the complexity of each of the complete single locus pedigree is linear, depending on the number of trios in the pedigree (Figure 2).

### Effect of population substructure and mutation

**Figure 3:**
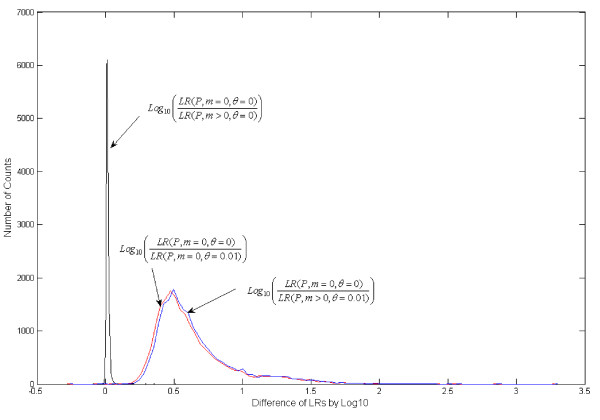
**Family pedigrees with mutations**. Comparison of log of pedigree (p) likelihood ratios for cases with and without mutation (m) and with and without population substructure (θ) given true identity (20,000 simulations). Mutation rate data are from the AABB [29].

Using the approaches described above to reduce complexity, an example (Figure 2) is provided to demonstrate how population substructure and mutation affect computational complexity. In Figure 2, several family members (F, C_1 _and C_2_) are typed to assess if the genotype of an unidentified individual (U) is consistent with the biological relationship of the missing person. In total, 20,000 Caucasian families of such a pedigree were simulated for the 13 STR CODIS loci [34], then LRs were calculated with a co-ancestry coefficient of 0.01 and mutation rates as published previously [29]. Table 1 shows that both population substructure and mutation substantially increase the time of the computation due to complexity. The effect of population substructure is moderate, which is mainly due to the one OSTP (U) in this pedigree, that is, computational time doubles at most. By contrast, mutation markedly increases computation time, because the *NGUC *of each UC (S for H_p_; U and S for H_d_) boosts, depending on the genotypes of the typed individuals, the pedigree structure and the number of alleles at each locus. Empirically, the cumulative effects of both factors can increase the computation time by one or more orders of magnitude (Table 1; Figure 3).

**Table 1:** Running time (seconds) for 20,000 simulations

	Co-ancestry coefficient	
	
	*θ *= 0	*θ *= 0.01
No mutation	1,140	1,344

With mutation	6,893	12,946

The likelihood ratios are also compared in the absence and presence of population substructure, with and without mutation by the same simulated pedigrees used above (Figure 3). The differences of logarithms of pedigree LRs [Log_10_(LR)] were plotted. Differences in Log_10_(LR)s in the absence and presence of mutation are usually < 0.1. Clearly, mutation has a limited effect on the LR ratio, because of the relatively low probability of mutation in a single case scenario. Often LRs with the mutation model are slightly lower than those that do not consider a mutation model, because the transmission probabilities between two identical alleles with the mutation model are < 1. However, incorporation of mutation is necessary to avoid possible false exclusions if mutations do exist in pedigrees. Population substructure substantially decreases the LRs, mostly from 2 to 100 times less than a scenario in which the absence of population substructure is assumed, as in this example. In other words, population substructure has a notable effect on the LR, and should be included in most identification cases.

### Mutation model

Dawid *et al. *[35] described a mutation model for autosomal STR loci. This model related the transmission probability between alleles to allele frequency, which is not supportable. The suggested factors that are related to STR loci mutation include repeat number, repeat motif, length of the repeat unit and flanking sequence, but not the allele frequency [16,17]. Other mutation models, which are uniform and proportional to the allele frequency, have also been proposed in some cases [15,36]. The decreasing model used in Familias [15] also does not appear to be supportable. This model includes a parameter (the number of 'possible' alleles) that cannot be determined. In addition, mutation probability is not related to the number of possible alleles [16,17]. In our study, we used the Two Phase Model because it is the most realistic mutation model of those defined for microsatellite loci [25]. This model does not limit the number of alleles at a single locus. The number of observed alleles only affects the inference of genotypes of untyped individuals in the reference family. Because the summation of equation (8) is always equal to 1 (equation 11), this model allows for an unlimited number of alleles, which is different from the mutation model used in Familias.

(11) $$\sum_{x=1}^{\infty }\alpha {\left(1-\alpha \right)}^{x-1}=\alpha \left[1+\frac{1}{1-\alpha }+\frac{1}{{\left(1-\alpha \right)}^{2}}+...\right]=1$$

The probabilities of gaining or losing steps depend on the number of steps between alleles [37]; for now equal mutation rates are assumed for gaining or losing steps in our model, but this can be adjusted if desired, which may be necessary as additional data on STR mutation patterns are developed. Currently, silent/null alleles are not considered because it is difficult to determine if a null allele exists in a complex pedigree with multiple untyped individuals.

### Allele frequency

A minimum allele frequency rule was adopted to accommodate identity-testing requirements. If the frequency of an allele is < 5/2n (a threshold value supported by Budowle *et al. *[38], with n being the sample size of the locus), then the frequency of the allele will be automatically raised to 5/2n. Invoking a minimum allele frequency threshold will result in the sum of the allele frequencies being > 1, which in turn will make the LR more conservative than other approaches. For example, Familias [15] normalized the allele frequencies so that the sum of the allele frequencies is 1, which may change the allele frequencies slightly and lead to different LR results compared with a minimal allele frequency correction approach (see Additional file 1). If the same allele frequencies are used and the sum of allele frequencies is 1 for a locus, Familias produces the same LR as MPKin in the absence of mutations.

### Validation

The software MPKin was validated in part with assistance from the International Commission on Missing Persons (ICMP). LRs of each locus of three pedigrees calculated by DNAView, Familias and MPKin were identical. Familias and MPKin can further calculate LRs accommodating population substructure and mutation. Familias may also give LR with mutations, but the mutation models used in Familias are not applicable to human STRs (see Additional file 1).

## Conclusion

In summary, this study provides a descriptive approach to assist the forensic DNA community in person identification for complex forensic identity and paternity testing cases. This process evaluates the putative biological relationships of individuals by calculating and ranking pedigree LRs for multiple putative pedigrees. Currently, this approach does not address linked markers because the linkage adjustments currently are unnecessary for forensic autosomal STRs. Adjustments for population substructure and mutation are incorporated, and LR values can be provided for any pedigree even with multiple large-step mutations. There is no limit on pedigree structure (for instance, incest can be addressed) and number of family members. However, because of the complexity of computation, incorporating multiple UCs can take time; for instance, MPkin can accommodate up to 4-5 UCs with population substructure and mutation in a reasonable time (several hours for a 13-STR pedigree with 4-5 UCs). The computation time can be decreased by ignoring large-step mutations. Fortunately, most forensic cases do not exceed this limitation. Lastly, the process described here can accommodate autosomal loci, Y chromosome haplotypes and mtDNA haplotypes. Independence across these three genetic marker systems is assumed.

## Competing interests

The authors declare that they have no competing interests.

## Authors' contributions

JG designed the algorithm, developed the software program and wrote most of the manuscript. BB and RC supervised this study and helped revising the manuscript. All the authors read and approved the manuscript.

## Supplementary Material

Additional file 1 **Validation of MPKin**. Three pedigrees were simulated for 13 CODIS loci, Penta D and Penta E according to USA Caucasian allele frequencies from STRBase [34]. As can be seen from the following three examples, MPKin yields the same LRs as those of DNAView in the absence of both population substructure and mutation. MPKin can further calculate LRs with both population substructure and mutation incorporated. Generally, LRs with either or both factors are reduced, which is consistent with the simulation study above. Click here for file
