# Supplementary material for: DNA identification by pedigree likelihood ratio accommodating population substructure and mutations
Source: Investig Genet. 2010 Oct 4;1:8. doi: 10.1186/2041-2223-1-8 (PMC2990736; doi:10.1186/2041-2223-1-8)
Supplement: Additional file 1 — Validation of MPKin. Three pedigrees were simulated for 13 CODIS loci, Penta D and Penta E according to USA Caucasian allele frequencies from STRBase [34]. As can be seen from the following three examples, MPKin yields the same LRs as those of DNAView in the absence of both population substructure and mutation. MPKin can further calculate LRs with both population substructure and mutation incorporated. Generally, LRs with either or both factors are reduced, which is consistent with the simulation study above. [file 2041-2223-1-8-S1.DOCX]

**Appendix: Validation of MPKin**

Three pedigrees were simulated for 13 CODIS loci, Penta D and Penta E according to USA Caucasian allele frequencies from STRBase [34]. As you can see from the following three examples, MPKin yields the same LRs as those of DNAView in the absence of both population substructure and mutation. MPKin can further calculate LRs with both population substructure and mutation incorporated. Generally, LRs with either or both factors are reduced, which is consistent with the simulation study above.

1. Pedigree-1

In the pedigree shown in Figure A1 (same as Figure 2), C1 and C2 are the alleged children of U, F is the alleged father, M is the alleged mother, and S is alleged spouse. M and S are not typed for autosomal STRs, but M is typed for mtDNA. The question is whether U belongs to the pedigree or U is unrelated to the pedigree. Table A1.1 gives the LRs from MPKin (in the absence and presence of population substructure and mutation) and DNAView for autosomal STRs; Table A1.2 gives the haplotype frequencies for Y STRs; Table A1.3 gives the haplotype frequencies for mtDNA.

Figure A1. Pedigree to identify the missing person U. M is only typed for mtDNA.

U

**?**

F

M

S

C_1_

C_2_

- 1. Autosomal STRs

Table A1.1. Likelihood ratios of MPKin, Familias (in the absence and presence of population substructure and mutation) and DNAView for pedigree-1. Mutation model “Prob. decreasing with range (stable)” with mutation range = 0.1 across all loci was used in Familias.

| Marker | Likelihood Ratios | | | | | | | | |
| --- | --- | --- | --- | --- | --- | --- | --- | --- | --- |
|  | DNAView | MPKin | MPKin with θ=0.01 | MPKin with mutation | MPKin with θ=0.01 and mutation | Familias | Familias with θ=0.01 | Familias with mutation | Familias with θ=0.01 and mutation |
| CSF1PO | 1.76 | 1.76 | 1.8 | 1.76 | 1.79 | 1.87 | 1.89 | 1.86 | 1.89 |
| D16S359 | 28.1 | 28.1 | 18 | 28.1 | 18 | 28.72 | 17.58 | 28.71 | 17.57 |
| D7S820 | 2.6 | 2.6 | 2.47 | 2.6 | 2.47 | 2.64 | 2.57 | 2.64 | 2.56 |
| D13S317 | 6.01 | 6.01 | 5.63 | 6.01 | 5.62 | 6.12 | 5.37 | 6.12 | 5.37 |
| D5S818 | 7.46 | 7.46 | 7.68 | 7.46 | 7.68 | 7.69 | 6.35 | 7.67 | 6.33 |
| D3S1358 | 1.29 | 1.29 | 1.16 | 1.3 | 1.16 | 1.36 | 1.17 | 1.36 | 1.17 |
| D8S1179 | 11 | 11 | 10.2 | 10.9 | 10.1 | 11.53 | 10.48 | 11.50 | 10.43 |
| D18S51 | 15.3 | 15.3 | 14.6 | 14.7 | 14 | 15.72 | 14.98 | 15.30 | 14.54 |
| D21S11 | 5.51 | 5.51 | 4.69 | 5.5 | 4.68 | 5.85 | 4.88 | 5.85 | 4.88 |
| FGA | 0.818 | 0.818 | 0.734 | 0.828 | 0.745 | 0.892 | 0.784 | 0.892 | 0.784 |
| VWA | 6.99 | 6.99 | 6.72 | 6.92 | 6.65 | 7.43 | 7.10 | 7.39 | 7.07 |
| TPOX | 3.66 | 3.66 | 3.59 | 3.66 | 3.59 | 3.79 | 3.70 | 3.79 | 3.70 |
| TH01 | 22.2 | 22.2 | 21.3 | 22.2 | 21.3 | 23.02 | 21.32 | 23.02 | 21.31 |
| PENTAD | 4.71 | 4.71 | 4.47 | 4.68 | 4.44 | 4.97 | 4.19 | 4.97 | 4.19 |
| PENTAE | 2.48 | 2.48 | 2.27 | 2.48 | 2.27 | 2.57 | 2.33 | 2.56 | 2.33 |
| Total | 3.74E+10 | 3.74E+10 | 1.07E+10 | 3.57E+10 | 1.01E+10 | 7.06E+10 | 1.12E+10 | 6.76E+10 | 1.07E+10 |

Note: DNAView gave the formulas for each locus, and LRs were calculated using the same allele frequencies as MPKin. Familias normalized the allele frequencies of each locus to make the sum of the frequencies to 1, which leads to slightly different LR results from those of MPKin. Familias can provide the same LRs as MPKin in absence of mutations using identical allele frequencies database, in which the sum of allele frequencies at each locus is one. Familias produces different LRs with mutations compared to MPKIN, since different mutation models were adopted in these two programs.

- 1. Y STRs

Table A1.2. Y STR Haplotype frequency for total and each population. The Y haplotype includes all 16 Y STRs in Yfiler.

| Population | Counts | Sample Size | Frequency | θ | Conditional frequency | CI(0.95) UpperBound |
| --- | --- | --- | --- | --- | --- | --- |
| Total | 1 | 7812 | 1.28E-04 | 9.34E-05 | 2.21E-04 | 7.13E-4 |
| African Ame. | 0 | 1439 | 0 | 4.77E-05 | 4.77E-05 | 2.56E-03 |
| Asian | 1 | 3018 | 3.31E-04 | 3.72E-04 | 7.03E-04 | 0.00184 |
| Caucasian | 0 | 1711 | 0 | 1.077E-04 | 1.08E-04 | **2.15E-03** |
| Hispanic | 0 | 730 | 0 | 9.23E-05 | 9.23E-05 | 5.04E-03 |

F, C1, C2 and U have the same Y haplotype. The transition probability from F to U is 0.9669868 with mutation included (mutation rates from [37] and STRBase). Hence, for Caucasian, the LR of Y haplotype is (0.9669868)^3^/2.15E-03 = 420.6.

- 1. Mitochondria DNA

Table A1.2. mtDNA Haplotype frequency for total and each population. The mtDNA includes both HV1 and HV2.

| Population | Sample  Size | Mismatch = 0 | | | Mismatch<=2 | | |
| --- | --- | --- | --- | --- | --- | --- | --- |
|  |  | Counts | Exact  frequency | CI(0.95)  UpperBound | Counts | General  frequency | CI(0.95)  UpperBound |
| Total | 5982 | 0 | 0 | 6.17E-04 | 28 | 0.0047 | 6.76E-03 |
| African Ame. | 1653 | 0 | 0 | 2.23E-01 | 1 | 6.05E-04 | 3.37E-01 |
| Asian | 937 | 0 | 0 | 3.93E-03 | 10 | 0.0011 | 0.0195 |
| Caucasian | 2116 | 0 | 0 | 1.74E-03 | 6 | 0.0028 | **0.0062** |
| Hispanic | 924 | 0 | 0 | 3.98E-01 | 1 | 0.0011 | 0.006 |

M and U have the same mtDNA haplotype. The transition probability from M to U is 1. Hence, for Caucasian, the LR of mtDNA haplotype is 1/0.0062= 161.3.

Combining autosomal STRs, Y STR haplotypes and mtDNA haplotypes together, with mutation and population substructure, the LR can reach 1.01E+10 *420.6 * 161.3 = 6.86E+14.

1. Pedigree-2

In the pedigree shown in Figure A2, F and M have daughter G1_D1; G1_D1 and her husband G1_D1H have a son G2_S1; G2_S1 and his wife G2_S1W have a son G3_S1; F and his daughter G1_D1 have a son G1_INC from an incest mating. Only G1_INC and M are typed. The question is whether the unknown person is consistent with G3_S1 belonging to the pedigree or is unrelated to the pedigree. Table A2 gives the LRs of MPKin (in the absence and presence of population substructure and mutation) and DNAView.

G1_D1

G1_INC

F

M

G1_D1H

G2_S1

?

G2_S1W

G3_S1

Figure A2. Pedigree to identify the missing person G3_S1.

Table A2. Likelihood ratios of MPKin (in the absence and presence of population substructure and mutation) and DNAView for pedigree-2. Familias provided the same LRs as MPKin in absence of mutations if the same allele frequencies were used, but different LRs with mutations because of different mutation models.

| Marker | Likelihood Ratios | | | | |
| --- | --- | --- | --- | --- | --- |
|  | MPKin | DNAView | MPKin with θ=0.01 | MPKin with mutation | MPKin with θ=0.01 and mutation |
| CSF1PO | 0.932 | 0.932 | 0.924 | 0.931 | 0.924 |
| D16S359 | 7.97 | 7.97 | 5.32 | 7.97 | 5.32 |
| D7S820 | 1.29 | 1.29 | 1.25 | 1.28 | 1.25 |
| D13S317 | 1.12 | 1.12 | 1.12 | 1.11 | 1.11 |
| D5S818 | 0.576 | 0.576 | 0.561 | 0.579 | 0.562 |
| D3S1358 | 1.37 | 1.37 | 1.37 | 1.37 | 1.37 |
| D8S1179 | 0.809 | 0.809 | 0.818 | 0.808 | 0.818 |
| D18S51 | 0.901 | 0.901 | 0.861 | 0.899 | 0.860 |
| D21S11 | 2.53 | 2.53 | 2.30 | 2.52 | 2.29 |
| FGA | 1.24 | 1.24 | 1.19 | 1.24 | 1.19 |
| VWA | 1.15 | 1.15 | 1.16 | 1.14 | 1.16 |
| TPOX | 0.50 | 0.50 | 0.488 | 0.50 | 0.489 |
| TH01 | 1.07 | 1.07 | 1.10 | 1.08 | 1.11 |
| PENTAD | 0.808 | 0.808 | 0.773 | 0.813 | 0.775 |
| PENTAE | 1.67 | 1.67 | 1.60 | 1.67 | 1.59 |
| Total | 16.03 | 16.03 | 7.85 | 15.86 | 7.81 |

1. Pedigree-3

In the pedigree shown in Figure A3, F and M have two typed non-fullsib great-grandchildren G3_S1 and G3_S2. The question is whether F is the great-grandfather of G3_S1 and G3_S2 or F and M are unrelated to them. Table A3 gives the LRs of MPKin (in the absence of mutation) and DNAView.

G1_S2

G1_S1

**?**

F

M

G1_S2W

G2_S1

G2_S2W

G3_S2

G3_S1

G1_S1W

G2_S2

G2_S1W

Figure A3. Pedigree to identify the missing person F.

Table A3. Likelihood ratios of MPKin (in the absence of mutation) and DNAView for pedigree-3. Familias provided the same LRs as MPKin in absence of mutations if the same allele frequencies were used, but different LRs with mutations because of different mutation models.

| Marker | Likelihood Ratios | | | | |
| --- | --- | --- | --- | --- | --- |
|  | MPKin | DNAView | MPKin with θ=0.01 | MPKin with mutation | MPKin with θ=0.01 and mutation |
| CSF1PO | 1.129 | 1.129 | 1.149 | 1.128 | 1.148 |
| D16S359 | 10.78 | 10.78 | 10.08 | 10.79 | 10.09 |
| D7S820 | 1.187 | 1.187 | 1.183 | 1.188 | 1.184 |
| D13S317 | 1.279 | 1.279 | 1.296 | 1.278 | 1.297 |
| D5S818 | 0.721 | 0.721 | 0.689 | 0.721 | 0.691 |
| D3S1358 | 1.673 | 1.673 | 1.722 | 1.672 | 1.722 |
| D8S1179 | 1.451 | 1.451 | 1.504 | 1.447 | 1.499 |
| D18S51 | 0.56 | 0.56 | 0.519 | 0.562 | 0.522 |
| D21S11 | 2.562 | 2.562 | 2.577 | 2.559 | 2.571 |
| FGA | 1.322 | 1.322 | 1.327 | 1.328 | 1.337 |
| VWA | 0.987 | 0.987 | 1.003 | 0.989 | 1.006 |
| TPOX | 0.567 | 0.567 | 0.524 | 0.567 | 0.524 |
| TH01 | 1.021 | 1.021 | 1.013 | 1.021 | 1.013 |
| PENTAD | 0.842 | 0.842 | 0.839 | 0.843 | 0.84 |
| PENTAE | 1.573 | 1.573 | 1.582 | 1.576 | 1.587 |
| Total | 46.42 | 46.42 | 39.75 | 46.87 | 40.53 |
